# The relationship between restrained eating and physical activity among female university students: a cross-lagged study

**DOI:** 10.3389/fnut.2026.1804133

**Published:** 2026-04-15

**Authors:** Chang Hu, Wenying Huang, Yan Liu

**Affiliations:** School of Physical Education, Jiangxi Normal University, Nanchang, China

**Keywords:** cross-lagged panel model, female university students, longitudinal study, physical activity, restrained eating

## Abstract

**Background:**

Restrained eating (RE) is common among female university students and is closely related to weight management and other health-related behaviors. Physical activity (PA), another key health behavior in this population, may both influence and be influenced by RE. However, existing evidence is predominantly cross-sectional, limiting understanding of their reciprocal longitudinal relationship.

**Objective:**

This study aimed to examine the bidirectional longitudinal associations between RE and PA among female university students and to test the longitudinal measurement invariance of both constructs across three waves.

**Methods:**

A three-wave longitudinal study was conducted among 813 female university students aged 18–22 years, with assessments administered at 3-month intervals over a 6-month period. RE was assessed using the Restraint subscale of the Dutch Eating Behavior Questionnaire (DEBQ), and PA was measured using the Physical Activity Rating Scale-3 (PARS-3). Descriptive statistics and Pearson correlation analyses were first conducted. Longitudinal measurement invariance of RE and PA was then examined across the three waves at the configural, metric, and scalar levels. Finally, cross-lagged panel models (CLPMs) were estimated within a structural equation modeling framework to test the bidirectional longitudinal associations between RE and PA.

**Results:**

Both RE and PA demonstrated acceptable longitudinal measurement invariance across the three waves and significant autoregressive stability over time. Cross-lagged analyses revealed significant bidirectional positive associations. Specifically, RE positively predicted subsequent PA from T1 to T2 (*β* = 0.229, *p* < 0.001) and from T2 to T3 (*β* = 0.164, *p* < 0.001), whereas PA positively predicted subsequent RE from T1 to T2 (*β* = 0.098, *p* < 0.01) and from T2 to T3 (*β* = 0.431, *p* < 0.001).

**Conclusion:**

RE and PA exhibited stable reciprocal longitudinal associations among female university students. These findings suggest that dietary restraint and physical activity may function as mutually reinforcing health-related behaviors in the context of weight and body-shape management. Future interventions should address both behaviors simultaneously to promote healthier and more sustainable behavioral regulation.

## 1 Introduction

Restrained eating (RE) refers to a pattern of eating behavior characterized by the deliberate restriction of energy intake, reduction in food consumption, or avoidance of high-calorie foods for the purpose of controlling body weight or body shape (1). The concept emerged from early investigations into the association between eating behavior and weight regulation (2). As research in this area has expanded, RE has been recognized as a common phenomenon among young adults, particularly university students (3). Previous studies have shown that approximately 52.8% of university students report high levels of restrained eating (4). Given its close associations with emotional experiences, body image, and health-related behaviors, RE has attracted increasing research attention in studies of university populations (5). Existing evidence suggests that restrained eating is relatively prevalent in higher education settings (6), and among female university students, it may be especially salient because of greater concern with body image and weight management.

In the Chinese context, the sociocultural environment may further intensify the tendency toward restrained eating among female university students. In recent years, thinness-oriented beauty ideals have become increasingly prominent in Chinese society, shaped by traditional appearance norms, rapidly expanding social media exposure, and peer comparison in everyday campus life (7, 8). These influences may heighten external pressure and internalized standards regarding body weight and shape, thereby increasing female students’ susceptibility to dieting and other restrictive eating behaviors (9). This issue is particularly important because female university students are situated at a developmental stage characterized by identity formation, heightened sensitivity to social evaluation, and increasing autonomy in lifestyle choices (10). As a result, they may be especially vulnerable to appearance-related social pressures and more likely to adopt maladaptive weight-control strategies. Against this background, examining RE in Chinese female university students is not only theoretically meaningful but also practically relevant for understanding health-related behavioral regulation in this population.

RE may have important implications for both physical and psychological health. On the one hand, chronic or excessive dietary restraint may lead to inadequate nutritional intake, metabolic dysregulation, and reduced physical vitality (11). On the other hand, it has also been associated with emotional distress, disordered eating risk, and lower subjective wellbeing (12). As a self-regulation-related behavioral pattern, RE may also exert complex influences on physical activity (PA) (13). For some individuals, weight-loss motivation associated with restrained eating may increase engagement in exercise as a means of enhancing energy expenditure and achieving body-shape goals (14). For others, insufficient energy intake, fatigue, and an increased physical burden may undermine exercise tolerance and reduce motivation to engage in PA (15). Previous studies have suggested that both contextual factors, such as body-related pressure, media exposure, and stressful life events, and individual factors, such as body image, self-control, and emotion regulation strategies, may shape restrained eating and related health behaviors among female university students (16–18).

PA represents another key health-related behavior among university students. However, contemporary university populations generally exhibit insufficient PA, prolonged sedentary behavior, and imbalanced exercise participation patterns (19, 20). These trends affect not only body weight and physical fitness but are also closely related to sleep quality, emotional regulation, and broader health outcomes (21, 22). Among female university students in particular, academic stress, irregular daily schedules, low exercise self-efficacy, and heightened concerns about thinness and body-shape management may increase the likelihood of physical inactivity or foster exercise participation that is predominantly goal-driven, especially for weight control (23, 24). Such patterns may entail both physical and psychological risks. Accordingly, PA has become an important entry point for understanding health-related behavior in female university students.

From a public health perspective, PA is commonly associated with health, self-discipline, and the attainment of an ideal body shape (25). Regular exercise is widely acknowledged to improve cardiorespiratory fitness, metabolic function, and perceived health status, and is therefore often considered inherently health-promoting (26, 27). Nevertheless, public discourse tends to emphasize its positive effects on physical health and appearance management while overlooking the complexity of PA in psychological and behavioral regulation. In practice, PA may serve different functions: it may be undertaken for enjoyment and health promotion, but it may also be used to compensate for food intake, alleviate guilt, or pursue short-term weight-loss goals (28). As a consequence, variation in exercise motivation and exercise patterns may lead to markedly different health outcomes. Moreover, individuals’ psychological state and perceived health may also influence their engagement in PA (29). In general, those experiencing more positive affect and greater perceived life control are more likely to participate in PA (30), whereas elevated stress, negative mood, and body image concerns may either reduce activity levels or foster more compulsive and compensatory forms of exercise (31, 32). Therefore, PA is not only an important predictor of health outcomes but also a central component of the broader system of health-related behaviors.

RE and PA are two of the most common behaviors involved in weight and body-shape management among female university students, and they may be closely interconnected through mechanisms of energy balance, body regulation, and self-control (33). Existing research suggests that under the influence of healthism, self-discipline discourse, weight stigma, and appearance-based evaluation, female university students may experience elevated body-related pressure and adopt weight-control strategies such as restrained eating (34). At the same time, PA is widely regarded as an effective means of body-shape management (35, 36). From one perspective, RE may prospectively promote PA. When individuals are strongly motivated by weight-loss goals, dietary restraint may be accompanied by increased exercise participation to enhance energy deficit and accelerate changes in body shape or body weight (37, 38). From another perspective, however, RE may also inhibit PA. Prolonged or excessive restriction of energy intake may lead to fatigue, reduced concentration, impaired recovery, and lower exercise tolerance, thereby weakening exercise adherence and resulting in lower or more unstable PA levels (39). In addition, RE is often associated with negative affect, body dissatisfaction, and depletion of self-regulatory resources, all of which may contribute to dysfunctional compensatory patterns between eating and exercise (40). For example, some individuals may increase PA after eating to offset guilt, whereas others may further intensify dietary restraint while reducing PA during periods of stress or emotional exhaustion (41).

Conversely, PA may also influence RE. Previous studies have indicated that PA among female university students is often linked to goals related to weight control and body-shape improvement (42). In this context, higher levels of PA may reinforce self-imposed expectations for thinness and intensify the perceived need to regulate eating, thereby increasing the likelihood of restrained or otherwise controlled eating strategies (12). However, PA does not inevitably promote dietary restraint. Regular participation in PA has also been shown to improve emotional wellbeing, enhance body satisfaction and self-efficacy, and foster more adaptive health cognitions and self-regulatory capacities (43). Through these pathways, PA may reduce excessive restraint tendencies driven by body-related pressure or negative emotional states, thereby promoting more stable and sustainable eating behaviors (44). Furthermore, the association between PA and RE may vary depending on exercise motivation and subjective experience. Some individuals may tightly couple exercise with dietary control through compensatory beliefs, whereas others may become less reliant on restrained eating because of the stress-buffering effects and positive bodily experiences associated with exercise (45). These findings indicate that PA may also have both positive and negative prospective effects on RE.

Despite growing interest in eating behavior and exercise among young women, research directly examining the relationship between RE and PA remains limited. More importantly, existing studies have largely relied on cross-sectional designs (46). Although cross-sectional research can identify associations between variables at a single point in time, it cannot establish temporal precedence or reciprocal predictive effects, nor can it adequately capture the dynamic interplay between RE and PA over time (47). A longitudinal design is therefore needed to clarify whether RE predicts later PA, whether PA predicts later RE, and whether these associations are bidirectional.

### 1.1 The present study

Against this background, the present study adopts a longitudinal design to examine the dynamic relationship between RE and PA. Although the sample was drawn from China, thinness-oriented beauty ideals, social media-driven appearance norms, and peer comparison have increasingly shaped young women’s concerns about body weight and body shape across diverse cultural contexts. Therefore, investigating the relationship between RE and PA is important not only for understanding health behavior patterns in a specific sample, but also for elucidating broader mechanisms underlying weight-management-related behaviors among young women. Using a cross-lagged panel model, this study examines the temporal associations and reciprocal predictive effects between RE and PA.

**Figure 1:**
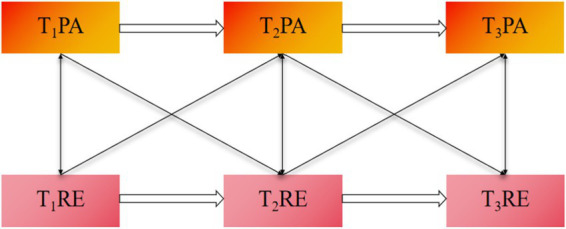
Hypothesized cross-lagged model of the reciprocal relationship between RE and PA.

The rationale for this investigation is grounded in the possibility that RE and PA are linked through multiple behavioral and psychological mechanisms. On the one hand, RE may promote PA through weight-control motivation, but it may also inhibit PA through energy depletion, fatigue, and emotional burden. On the other hand, PA may reinforce RE when exercise is embedded in compensatory or appearance-driven regulation, but it may also reduce RE by improving emotional state, body satisfaction, and self-regulatory functioning. Based on these theoretical considerations and prior empirical evidence, the present study proposes the following hypothesis (see Figure 1):

*Hypothesis*: RE and PA are bidirectionally associated over time.

By clarifying the dynamic relationship between RE and PA, the present study seeks to enrich understanding of the interplay between weight-management-related health behaviors and to provide empirical support for future intervention and health promotion efforts targeting young women.

## 2 Materials and methods

### 2.1 Participants and procedure

The present study employed a three-wave longitudinal design over a six-month period, with two follow-up assessments at 3-month intervals. Before formal data collection, an *a priori* power analysis was conducted in G*Power 3.1 to obtain a rough minimum sample size estimate for repeated within-subject assessments. The analysis assumed *f* = 0.25, *α* = 0.05, 1 − *β* = 0.95, three measurement occasions, a correlation of 0.60 among repeated measures based on Hu et al. (38), and a nonsphericity correction of 1. The final sample size substantially exceeded this minimum threshold.

In addition, following the commonly used rule of thumb that the sample size for structural equation modeling should be at least 10 times the number of observed items, and given that the present study included 13 items, the minimum required sample size was estimated at 130. Assuming an attrition rate of 20% across the three waves, the minimum baseline sample size was set at 163 participants. To ensure adequate power and account for attrition, 1,068 questionnaires were collected at Wave 1, and valid cases were retained for subsequent analyses after excluding invalid responses.

A convenience sampling approach was used. To broaden sample coverage and minimize selection bias within practical constraints, we first conducted a stratified screening at the institutional level before recruitment. Specifically, accessible universities within the study region were first stratified according to institutional type (e.g., comprehensive vs. specialized universities) and campus context (e.g., urban vs. suburban/new district campuses). Subsequently, universities with administrative approval and feasible conditions for on-site data collection were selected from each stratum to participate. The final sample was drawn from 11 universities of different types and campus environments within Jiangxi Province, thereby capturing, to some extent, heterogeneous academic and living contexts. This stratified convenience sampling strategy enabled the inclusion of a more heterogeneous sample, providing reasonable diversity in academic settings and living backgrounds, which may be related to health behaviors such as physical activity and eating patterns.

After obtaining institutional approval, on-site group assessments were conducted among female university students. To minimize potential interference from vacations or major academic events, all three waves of data collection were scheduled on weekdays during regular class or self-study periods, avoiding examination weeks and periods immediately before or after national holidays. The specific data collection periods were as follows: Wave 1 (T1: March 15–19, 2025), Wave 2 (T2: June 13–16, 2025), and Wave 3 (T3: September 14–18, 2025). The 3-month interval was selected to balance the need to detect meaningful behavioral changes over time with the practical goal of reducing participant burden and attrition. All assessments were administered in classroom settings by trained research assistants following standardized procedures. Participants completed the questionnaires individually in a quiet, distraction-free setting. Before participation, the study aims and procedures were explained, and written informed consent was obtained. Participants were further informed that participation was voluntary and anonymous, and that they were free to withdraw at any time without penalty. To facilitate matching responses across the three waves, each participant was given a unique identification code. Following each wave, the data were screened for missingness and response quality (e.g., patterned responding) before subsequent analyses were conducted.

**Figure 2:**
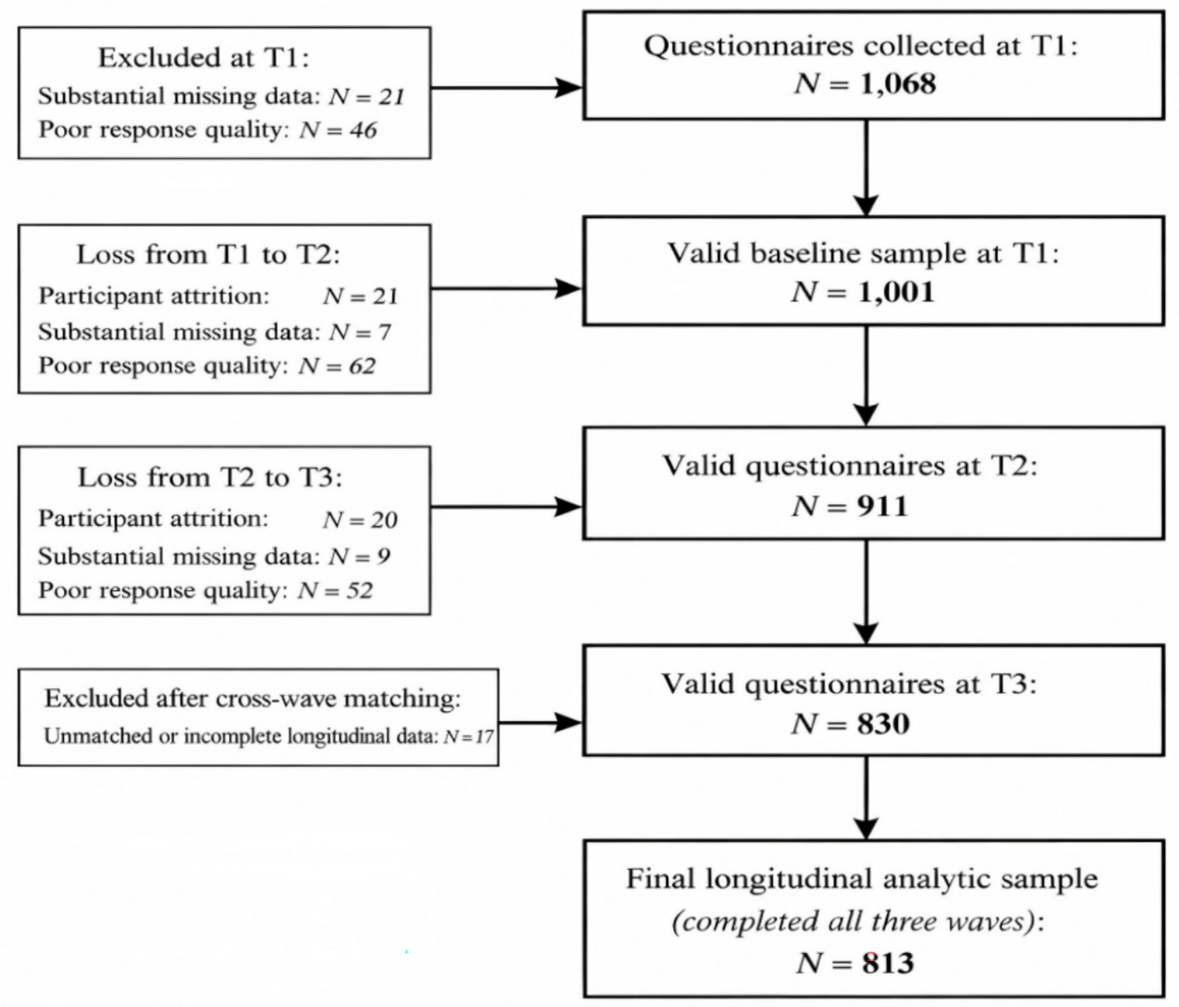
Recruitment and retention of participants.

At T1, 1,068 questionnaires were collected, of which 1,001 were considered valid and used as the baseline sample. At T2, 980 questionnaires were returned and 911 were deemed valid. At T3, 891 questionnaires were collected, including 830 valid cases. Questionnaires were excluded if they contained substantial missing data, showed poor response quality (e.g., obvious patterned responding), involved duplicate submissions, or included invalid or unmatchable identification codes. After data-quality screening and cross-wave matching, 813 participants who provided valid data at all three waves were retained as the final longitudinal analytic sample. Details of participant recruitment and retention are provided in Figure 2.

Among these participants, 363 were from urban areas (44.60%) and 450 were from rural areas (55.40%). The sample included 352 first-year students, 335 s-year students, and 126 third-year students. At T1, participants ranged in age from 18 to 22 years ( M=19.96 , SD=1.43 ). Sample characteristics are presented in Table 1.

**Table 1:** Demographic characteristics of the participants (*N* = 813).

Characteristic	*n* (%)/M ± SD
Age, years	19.96 ± 1.43
Age range	18–22
Residential background	
Urban	363 (44.60)
Rural	450 (55.40)
Academic year	
First-year	352 (43.30)
Second-year	335 (41.21)
Third-year	126 (15.50)

To evaluate the possibility of systematic attrition, a dichotomous variable labeled data completeness was created (0 = complete data across all three waves; 1 = missing data at one or more waves). Based on the 1,001 valid baseline cases, 813 participants were classified into the complete-data group and 188 into the incomplete-data group, corresponding to an attrition rate of 18.78%. Chi-square tests further showed that the two groups did not differ significantly in academic year (*χ^2^* = 3.21, *p* = 0.07) or residential background (*χ^2^* = 1.58, *p* = 0.21). Independent-samples *t*-tests based on T1 data further revealed no significant group differences in PA (*t* = −0.81, *p* = 0.42) or RE (*t* = 1.06, *p* = 0.29). Taken together, these findings indicate that attrition was not systematically related to academic year, residential background, or the main study variables (PA and RE) in the present sample. All procedures were reviewed and approved by the Institutional Review Board of Jiangxi Normal University (IRB-JXNU-PEC-20240106).

### 2.2 Measures

#### 2.2.1 Restrained eating

RE was assessed using the Restraint subscale of the Dutch Eating Behavior Questionnaire (DEBQ) (48). The Chinese version translated and psychometrically validated by Wu et al. (86), was administered in this study. The subscale consists of 10 items rated on a 5-point Likert scale, with higher scores indicating greater restrained eating. A sample item is: “If you gain weight, do you eat less than usual in order to lose weight?” In the present study, the subscale demonstrated excellent internal consistency across the three waves (Cronbach’s *α* = 0.906 at T1, 0.914 at T2, and 0.896 at T3).

#### 2.2.2 Physical activity rating scale

PA was assessed using the Physical Activity Rating Scale-3 (PARS-3), revised by Liang (49). The scale measures physical activity participation across three dimensions: exercise intensity, duration, and frequency. Each dimension is rated on a 5-point scale. The total PA score is calculated as intensity × (duration − 1) × frequency, yielding a possible range of 0 to 100, with higher scores indicating greater physical activity participation. Based on established cutoff criteria, participants can be classified into low (≤19), moderate (20–42), and high (≥43) levels of physical activity. The PARS-3 has been widely used among Chinese university students. In the present study, the scale showed acceptable internal consistency across the three waves (Cronbach’s *α* = 0.749 at T1, 0.755 at T2, and 0.747 at T3).

### 2.3 Data analysis

Data analyses were conducted in two stages. First, all valid questionnaire data from the three waves were entered into SPSS 26.0 for preliminary analyses. Descriptive statistics, including means, standard deviations, skewness, and kurtosis, were calculated for each variable at each time point, and Pearson correlation analyses were conducted to examine the bivariate associations between RE and PA across the three waves. In addition, Harman’s single-factor test was performed to provide a preliminary assessment of common method bias.

Second, prior to testing the longitudinal associations between RE and PA, the longitudinal measurement invariance of both constructs across the three waves was examined in Mplus 8.3. Following standard procedures, configural invariance, metric invariance, and scalar invariance were tested sequentially to determine whether the constructs were measured equivalently over time. Model fit was evaluated using *χ*^2^, degrees of freedom (*df*), the comparative fit index (CFI), the Tucker–Lewis index (TLI), the standardized root mean square residual (SRMR), and the root mean square error of approximation (RMSEA). Measurement invariance was considered acceptable when the changes in fit indices between adjacent nested models met conventional criteria, namely ΔCFI ≤ 0.01 and ΔTLI ≤ 0.01.

After establishing longitudinal measurement invariance, four competing cross-lagged panel models (CLPMs) were estimated in Mplus 8.3 to examine the reciprocal longitudinal relationship between PA and RE across the three waves. Model 1 included only autoregressive paths for PA and RE across adjacent waves. Model 2 added cross-lagged paths from PA to subsequent RE. Model 3 added cross-lagged paths from RE to subsequent PA. Model 4 included reciprocal cross-lagged paths between PA and RE across adjacent waves. Age, academic year, and residential background were included as control variables in the final model. The autoregressive paths were used to estimate the temporal stability of each construct over time, whereas the cross-lagged paths were used to test whether one construct prospectively predicted changes in the other at subsequent waves.

## 3 Results

### 3.1 Common method bias test

To assess the potential influence of common method bias, Harman’s single-factor test was conducted separately for the T1, T2, and T3 data. The results of the exploratory factor analyses indicated that two factors with eigenvalues greater than 1 were extracted at each wave. The first unrotated factor accounted for 40.964% of the total variance at T1, 43.011% at T2, and 38.390% at T3, all of which were below the commonly used cutoff of 50% (50). These findings provide preliminary evidence that common method bias was unlikely to seriously threaten the validity of the study results.

### 3.2 Descriptive statistics and correlations

Table 2 presents the descriptive statistics and Pearson correlation coefficients for all study variables. Normality tests indicated that the absolute values of skewness and kurtosis for all measures were within acceptable ranges, supporting the assumption of approximate normality. Correlation analyses showed that PA was significantly and positively associated with RE at each measurement occasion. Moreover, both constructs showed significant positive correlations across both adjacent and non-adjacent waves, indicating relative temporal stability. This pattern provided a basis for proceeding with the longitudinal cross-lagged analyses.

**Table 2:** Correlations and descriptive statistics for PA and RE across three time points.

Correlations	M	SD	Skewness	Kurtosis	T1PA	T2PA	T3PA	T1RE	T2RE	T3RE
T1PA	26.29	27.27	0.95	−0.26	1					
T2PA	23.37	26.85	1.14	0.19	0.392**	1				
T3PA	26.33	27.16	0.99	−0.02	0.401**	0.421**	1			
T1RE	3.039	0.99	0.01	−0.82	0.332**	0.332**	0.318**	1		
T2RE	3.188	1.04	−0.28	−0.92	0.282**	0.278**	0.269**	0.584**	1	
T3RE	2.739	0.96	0.47	−0.51	0.444**	0.483**	0.428**	0.500**	0.305**	1

Correlations are Pearson’s *r*. Significance: **p < 0.01.

### 3.3 Measurement invariance analysis

Before examining the longitudinal relationships among the primary variables, longitudinal measurement invariance of the PA and RE scales across the three measurement waves was first established. A set of nested models was compared to test configural, metric, and scalar invariance in a stepwise manner.

As presented in Table 3, the initial configural models for both constructs fit the data well, indicating that their factor structures were consistent across time. Next, equality constraints were introduced incrementally to assess more stringent levels of invariance. For PA, constraining factor loadings to be equal across time (M2 vs. M1: ΔCFI = 0.000, ΔTLI = 0.004) resulted in negligible changes in model fit. Further constraining item intercepts to equality (M3 vs. M2: ΔCFI = −0.008, ΔTLI = −0.009) also led to only minor changes, which remained within commonly accepted thresholds (|ΔCFI| ≤ 0.01, |ΔTLI| ≤ 0.01). Similarly, for RE, imposing equality constraints on factor loadings (M2 vs. M1: ΔCFI = −0.001, ΔTLI = 0.000) did not substantially deteriorate model fit. Additional constraints on item intercepts (M3 vs. M2: ΔCFI = 0.000, ΔTLI = 0.000) likewise resulted in no meaningful degradation in fit indices. Taken together, these findings support configural, metric, and scalar invariance of both PA and RE across the three time points.

**Table 3:** Testing for longitudinal invariance of data from three surveys.

Variable	Model	*χ^2^/df*	CFI	TLI	SRMR	RMSEA	Model comparison	ΔCFI	ΔTLI
PA	M1	0	1	1	0	0			
	M2	0.320	1.000	1.000	0.009	0.000	M2-M1	0.000	0.000
	M3	2.763	0.992	0.991	0.026	0.047	M3-M2	−0.008	−0.009
RE	M1	1.309	0.997	0.996	0.015	0.019			
	M2	1.335	0.996	0.996	0.024	0.020	M2-M1	−0.001	0.000
	M3	1.323	0.996	0.996	0.027	0.020	M3-M2	0.000	0.000

M1, configural model; M2, metric model; M3, scalar model; PA, physical activity; RE, restrained eating.

### 3.4 Cross-lagged analysis

To examine the longitudinal relationship between RE and PA, four competing cross-lagged panel models were estimated. M1 included only autoregressive paths, M2 added cross-lagged paths from PA to subsequent RE, M3 added cross-lagged paths from RE to subsequent PA, and M4 included reciprocal cross-lagged paths between RE and PA. As shown in Table 3, M1, M2, and M3 all showed poor fit to the data. In comparison, M4 demonstrated a substantially better fit than the other models. Therefore, M4 showed a clear improvement in fit over the alternative models and was therefore retained as the final model (see Table 4).

**Table 4:** Model fit indices for the four cross-lagged panel models.

Path	*χ^2^*	*df*	CFI	TLI	SRMR	RMSEA
M1	406.575	8	0.581	0.267	0.173	0.248
M2	246.281	6	0.748	0.411	0.143	0.222
M3	318.907	6	0.671	0.233	0.140	0.253
M4	19.623	4	0.822	0.876	0.078	0.079

**Figure 3:**
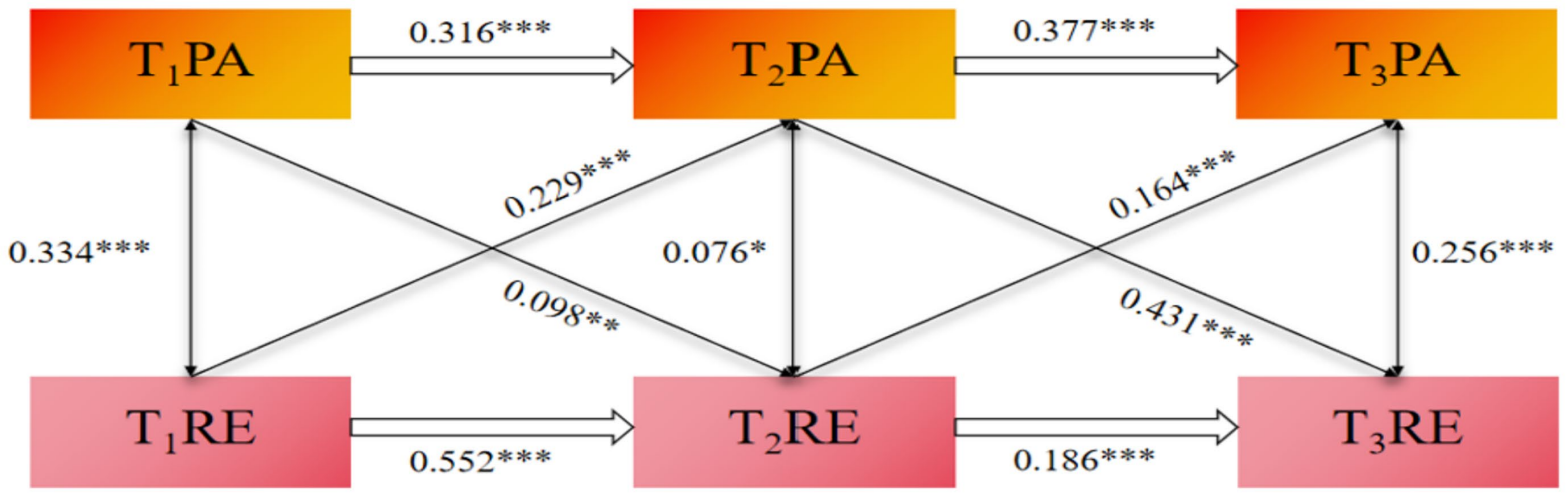
Standardized path coefficients of the three-wave cross-lagged model between PA and RE. *p < 0*.05*, **p < 0.01, ***p < 0*.001.*

Figure 3 presents the standardized path coefficients for Model M4, in which age, academic year, and residential background were included as control variables. Regarding autoregressive effects, PA at T1 significantly and positively predicted PA at T2 (*β* = 0.316, *p* < 0.001), and PA at T2 significantly and positively predicted PA at T3 (*β* = 0.377, *p* < 0.001) likewise, RE at T1 significantly and positively predicted RE at T2 (*β* = 0.552, *p* < 0.001), and RE at T2 significantly and positively predicted RE at T3 (*β* = 0.186, *p* < 0.001). These findings indicate that both constructs exhibited temporal stability across the three measurement waves. Second, regarding cross-lagged effects, PA showed significant positive prospective associations with subsequent RE. More specifically, PA at T1 was a significant positive predictor of RE at T2 (*β* = 0.098, *p* < 0.01), and PA at T2 likewise positively predicted RE at T3 (*β* = 0.431, *p* < 0.001). Significant positive effects were also observed in the opposite direction: RE at T1 predicted PA at T2 (*β* = 0.229, *p* < 0.001), and RE at T2 predicted PA at T3 (*β* = 0.164, *p* < 0.001). Overall, PA and RE exhibited robust bidirectional positive associations across the three measurement occasions, thus supporting the study hypothesis.

## 4 Discussion

The descriptive statistics of the present study indicated that PA levels among female university students remained relatively consistent across the three measurement waves, showing a generally stable pattern over time. This finding is broadly consistent with previous research reporting relative stability in physical activity patterns among university students and young adults, suggesting that individuals’ daily activity behaviors do not usually fluctuate substantially over short follow-up periods (51). One possible explanation is that academic and daily routines during university life, such as course schedules, extracurricular activities, and commuting patterns, tend to be relatively fixed, which may facilitate the formation of habitual physical activity behaviors that remain stable over time (52).

In contrast, RE showed greater fluctuation across the three waves, which may be related to the sociocultural context and developmental characteristics of Chinese female university students (3). On the one hand, thin-ideal beauty norms, peer comparison, and body-related content on social media may continuously reinforce pressure to manage body weight and shape (53–55). On the other hand, heightened sensitivity to external evaluation and social expectations within a collectivistic cultural context may further strengthen the tendency toward dietary control (56).

In addition, during the university period, individuals are confronted with multiple developmental tasks, including identity exploration, relationship formation, and academic and career pressures. These demands may make appearance-related self-worth and perceived control more vulnerable to fluctuation, thereby rendering restrained eating more sensitive to situational influences (57, 58). It should be noted that RE, as measured in the present study by the DEBQ, primarily reflects a tendency toward dietary self-regulation for weight management rather than extreme dieting or clinically diagnosed eating disorders.

The present study also found that RE positively predicted subsequent PA. Previous longitudinal research has similarly suggested that diet-related control behaviors may influence later patterns of exercise participation, particularly in contexts where weight and body shape management are salient goals (59). According to health behavior models, individuals’ engagement in health-related behaviors is jointly shaped by their perceptions of behavioral benefits and barriers, self-regulatory capacity, and behavioral intentions, which together determine how individuals invest in physical activity and derive psychological and behavioral outcomes (20, 60). Higher levels of restrained eating often reflect stronger health- or appearance-oriented self-management tendencies (2). Within this goal framework, individuals may be more likely to perceive physical activity as a high-benefit behavior, such as facilitating energy expenditure, improving body shape, and enhancing self-image, thereby strengthening exercise intentions and adherence (23, 61, 62). In addition, planning and monitoring habits developed during dietary control may enhance individuals’ perceived self-regulatory capacity, enabling them to better maintain exercise behavior when facing barriers such as time constraints, fatigue, or environmental limitations (63). Consequently, restrained eating may promote greater subsequent physical activity by reinforcing health management goals, increasing perceived exercise benefits, and enhancing self-regulation and exercise self-efficacy among female university students (2, 9).

The results further demonstrated that physical activity positively predicted restrained eating, suggesting that higher levels of physical activity in this context were associated with greater engagement in dietary management (46). Previous studies have also indicated that, among populations with strong body shape management concerns, exercise and dietary control may co-occur as a combined behavioral pattern, in which individuals simultaneously increase energy expenditure and regulate intake to achieve weight or body shape goals (24, 64). From the perspective of the Conservation of Resources theory, individuals strive to acquire, maintain, and invest valued resources (65). When physical condition, fitness, or appearance-related self-evaluations are regarded as important resources, physical activity may be perceived as a form of resource investment aimed at improving fitness, enhancing body shape, and obtaining positive feedback (66). Within this framework, individuals with higher levels of physical activity may be more likely to sustain health management efforts and adopt dietary restraint as a complementary daily strategy to maintain or amplify the physical and psychological benefits derived from exercise (67). At the same time, feedback and comparison processes embedded in exercise contexts may increase expectations regarding body management outcomes, leading individuals to devote greater attention and effort to dietary control. In some cases, such investment may also impose behavioral burdens and further strengthen tendencies toward dietary restraint (68). Taken together, physical activity may promote subsequent restrained eating by enhancing investment in health management, accumulating self-regulatory resources, and engaging with body-shape–oriented goals among female university students (69).

The cross-lagged panel model results indicated a bidirectional predictive relationship between physical activity and restrained eating, suggesting that their association is not simply unidirectional or linear, but instead reflects a more complex and dynamic interplay Previous longitudinal studies have similarly observed reciprocal influences among health-related behaviors (70, 71), suggesting that exercise and dietary behaviors may function as both antecedents and outcomes over time, reflecting interconnections and self-reinforcing processes within behavioral systems (72).

Self-efficacy theory may help explain the bidirectional association observed in this study (73). Individuals’ beliefs in their ability to successfully perform specific health behaviors influence their motivation, emotional experiences, and behavioral choices (74). Higher levels of exercise and dietary self-efficacy are more likely to encourage active participation in physical activity and more structured dietary control.

In contrast, lower self-efficacy may lead to greater fluctuation or discontinuation, undermining behavioral stability (75). Specifically, positive experiences and successful execution during physical activity may strengthen beliefs in self-discipline and health management, thereby promoting more sustained dietary restraint; conversely, higher levels of dietary control may enhance goal clarity and action organization, facilitating subsequent planning and execution of physical activity (76). As such, physical activity and restrained eating may form a reciprocal feedback loop that jointly shapes the trajectories of health behaviors among female university students, highlighting the complexity of interactions among contextual, cognitive, and behavioral factors in health behavior change (77).

An additional finding from the cross-lagged panel model was that the within-wave correlations were stronger than the across-wave autoregressive associations (78). This pattern may suggest that shared contextual influences more strongly shape variables assessed at the same time point. In contrast, associations across time can be weakened by temporal variability, changing contexts, and random disturbances (79). Methodological and longitudinal research has similarly noted that as time intervals increase, the rank-order stability of traits or behaviors tends to decline, and autoregressive correlations often weaken, reflecting dynamic changes driven by natural development and environmental influences (61, 80).

Given the relatively long intervals between measurement waves in the present study, both physical activity and restrained eating were likely influenced by external factors such as changes in academic workload, examination periods, and schedule adjustments, seasonal and weather variations, social media exposure, and accessibility of campus exercise resources, resulting in lower cross-time stability compared to within-time associations (81). These findings suggest that the bidirectional relationship between physical activity and restrained eating is shaped not only by individual-level factors but also by broader environmental conditions and situational pressures (43). For example, self-efficacy and body image satisfaction may influence dietary control and exercise participation through social support and peer norms, while psychological resilience, coping strategies, and health motivation may further moderate the pathways linking the two behaviors (82, 83).

Overall, these results indicate that additional unmeasured variables, as well as potential mediating and moderating mechanisms, may exist beyond the current model (84). Future research may benefit from incorporating factors such as body image, media comparison, exercise and dietary self-efficacy, sleep, and stress levels to more comprehensively elucidate the dynamic coupling of these health-related behaviors over time (85).

### 4.1 Implications and limitations

Overall, the present study advances understanding of the relationship between physical activity and restrained eating among female university students at both theoretical and practical levels, and offers actionable implications for health promotion in university settings. Unlike previous studies that have primarily focused on the unidirectional effects of physical activity on dietary behaviors or weight management, the present findings indicate that RE and PA are reciprocally related over time.

They may also prospectively predict subsequent PA levels, indicating a bidirectional relationship between the two behaviors. This finding enriches theoretical perspectives on health behavior interdependence and behavioral clustering. It offers a new lens through which to understand the potential long-term influence of restrained eating on trajectories of exercise participation. Accordingly, future interventions should avoid treating exercise promotion and dietary management as isolated components and instead adopt integrated strategies that advance both simultaneously.

On the one hand, courses and campus-based programs can be implemented to foster regular PA habits and enhance exercise self-efficacy; on the other hand, guidance toward more scientific and sustainable dietary regulation should be provided to reduce unhealthy extreme restriction and weight-related anxiety. From a preventive perspective, efforts may prioritize improving nutrition literacy, enhancing body image, and reducing appearance-based comparison pressures, thereby preventing dietary control from evolving into maladaptive behavior patterns under stress.

Educators and practitioners may adopt dual-pathway intervention approaches that combine structured exercise planning and behavioral support with goal setting, self-monitoring, emotion regulation, and stress management training, ultimately strengthening individuals’ self-regulatory capacity and long-term adherence. Moreover, integrating environmental factors, such as peer support, family and campus health climates, accessibility of exercise facilities, and optimization of health information environments, may help establish a positive feedback loop in which PA and healthy dietary regulation mutually reinforce each other, thereby enhancing health behavior maintenance, social adjustment, and psychological wellbeing among female university students.

Despite its contributions, several limitations of the present study should be acknowledged. First, physical activity was primarily assessed using the Physical Activity Rating Scale–3 (PARS-3), a self-report measure that may be subject to recall bias and social desirability effects, potentially leading to inaccuracies in estimating exercise frequency, intensity, and duration. In addition, the scale places greater emphasis on structured physical exercise and provides more limited coverage of daily physical activities such as walking for transportation or household tasks. Future research may benefit from combining self-report measures with objective indicators, such as accelerometers, wearable devices, or smartphone-based data on steps, heart rate, and activity trajectories, supplemented by activity logs or intensive longitudinal data, to more accurately capture activity levels and their fluctuations.

Second, although the present study identified stable associations between exercise volume and restrained eating as measured by the restrained eating subscale of the DEBQ, it did not further differentiate specific types of exercise or participation contexts. Different forms of physical activity may be associated with distinct motivational structures and psychological mechanisms. For example, group-based and cooperative activities may promote maintenance of health behaviors through peer support, a sense of belonging, and shared goals. In contrast, exercise contexts that emphasize body shaping and appearance presentation may be more likely to intensify dietary restraint tendencies or impose greater behavioral burdens for some individuals. Future research could differentiate between organized and unorganized activities, competitive and recreational sports, and group-based and individual exercise to more clearly identify which forms of physical activity best support sustainable dietary self-management and to inform the development of more targeted campus health programs.

Third, although the three-wave longitudinal design with an approximately 6-month follow-up period offers advantages over cross-sectional approaches for temporal inference, the fixed, relatively long measurement intervals may be insufficient to capture the short-term dynamics of the two behaviors at daily or weekly timescales. At the same time, longer-term follow-up may reveal cumulative effects. Future research could incorporate more intensive tracking methods, such as ecological momentary assessment or diary designs, and extend follow-up durations when feasible to examine the robustness of bidirectional associations across multiple temporal scales. Fourth, although the present analyses controlled for age, academic year, and residential background, unmeasured confounding factors may still have influenced the observed relationships. Such factors may include body image, body dissatisfaction, weight control intentions, appearance comparison pressure, health motivation, self-control–related traits, and health norms and support within family and peer environments. Future studies could incorporate these variables and examine their potential mediating or moderating roles to enhance explanatory power and strengthen the robustness of conclusions. Analytically, longitudinal models that more clearly distinguish between stable between-person differences and within-person changes may also be considered.

Finally, the characteristics of the sample may constrain the generalizability of these findings. Participants were primarily female university students from a single region, most of whom were generally healthy and able to participate in daily activities; therefore, the conclusions may not readily generalize to female students from other regions, different types of universities, or different academic stages. Future research should broaden the sample base and incorporate heterogeneity by including populations from diverse geographic and educational contexts and, when possible, accounting for variations in health status to enhance the applicability and external validity of findings.

## 5 Conclusion

The present study extends existing research by demonstrating that physical activity and restrained eating are linked through a stable bidirectional positive process among female university students. Rather than functioning in isolation, the two behaviors appear to co-develop over time as part of an interconnected system of weight- and health-related self-regulation. These findings highlight the value of integrated interventions that target both exercise and dietary regulation to foster healthier and more sustainable behavioral outcomes.

## Data Availability

The original contributions presented in the study are included in the article/supplementary material, further inquiries can be directed to the corresponding authors.
